# Risk of adverse pregnancy outcomes in pregnant women with gestational diabetes mellitus by age: a multicentric cohort study in Hebei, China

**DOI:** 10.1038/s41598-023-49916-2

**Published:** 2024-01-08

**Authors:** Ting Zhang, Meiling Tian, Ping Zhang, Liyan Du, Xuyuan Ma, Yingkui Zhang, Zengjun Tang

**Affiliations:** 1 Department of Reproductive Medicine, Hebei Reproductive Health Hospital, Shijiazhuang, China; 2 Department of Obstetrics and Gynecology, Hebei General Hospital, Shijiazhuang, China; 3 Department of Obstetrics and Gynecology, Hebei Maternity Hospital, Intersection of Hongqi Street and Xinshi North Road, Shijiazhuang, 050000 Hebei China; 4 Department of Information Management, Hebei Center for Women and Children’s Health, Shijiazhuang, China; 5 Department of Graduate School, Hebei Medical University, Shijiazhuang, China

**Keywords:** Endocrine system and metabolic diseases, Endocrine system and metabolic diseases

## Abstract

Gestational diabetes mellitus (GDM) is an unique metabolic disorder that occurs during pregnancy. Both GDM and advanced age increase the risk of adverse pregnancy outcomes. This study used a GDM cohort study to investigate the role of age in the adverse pregnancy outcomes for pregnant women with GDM. From 2015 to 2021, 308,175 pregnant women were selected, and the data received from 22 hospitals by the Hebei Province Maternal Near Miss Surveillance System. There were 24,551 pregnant women with GDM that were divided into five groups by age (20–24, 25–29, 30–34, 35–39, 40–44 years old). Because the prevalence of adverse pregnancy outcomes was lower in pregnant women with GDM aged 25–29, they were used as a reference group (*P* < 0.05). Compared with GDM women aged 25–29 years, GDM women aged 35–44 years had a significant higher risk of cesarean delivery (aOR: 2.86, 95% CI 2.52–3.25) (*P* < 0.001), abnormal fetal position (aOR: 1.78, 95% CI 1.31–2.37) (*P* < 0.001), pre-eclampsia (aOR: 1.28, 95% CI 1.01–1.61) (*P* < 0.05), macrosomia (aOR: 1.25, 95% CI 1.08–1.45) (*P* < 0.05), and large for gestational age (LGA) (aOR: 1.16, 95% CI 1.02–1.31) (*P* < 0.05), GDM women aged 40–44 years had a higher risk of placenta previa (aOR: 2.53, 95% CI 1.01–6.35) (*P* < 0.05), anemia (aOR: 3.45, 95% CI 1.23–9.68) (*P* < 0.05) and small for gestational age (aOR: 1.32, 95% CI 1.01–1.60) (*P* < 0.05). Advanced maternal age was an independent risk factor for abnormal fetal position, pre-eclampsia, anemia, macrosomia, and LGA in pregnant women with GDM.

## Introduction

Gestational diabetes mellitus (GDM) is a kind of hyperglycemia that initially appears or is detected during pregnancy, which is one of the most common obstetric complications^1^. In 2017, the global prevalence of GDM was 9.0%^2^. From 2011 to 2018, the average prevalence of GDM in China was 14.8%, ranging from 5.1 to 33.3% in various locations^3^.

GDM is a risk factor for adverse maternal and perinatal outcomes^4^. These adverse pregnancy outcomes include pre-eclampsia, preterm birth, caesarean section, stillbirth, macrosomia, large for gestational age (LGA), respiratory distress syndrome, fetal malformations, neonatal hypoglycemia, and neonatal intensive care unit (NICU) admission^4–6^. Even from that, women with a history of GDM have an even greater risk to get type 2 diabetes mellitus, metabolic syndrome^7^ and cardiovascular diseases^8^. And their offspring are more likely to have metabolic illness^9^.

Advanced maternal age (AMA) is currently identified as a key risk factor for GDM and adverse pregnancy outcomes^10^. GDM is more common in women during pregnancy with AMA^11^. And AMA is related to the prevalence of stillbirth, preterm birth, small for gestational age (SGA), macrosomia^12^. The present situation in China is that the number of pregnant women with AMA and GDM has risen since the implementation of the two-child policy^12^. There will be more pregnant women in China with AMA and GDM as China's fertility strategy changes to encourage the birth of third children. Is there an impact of age on pregnancy outcomes of pregnant women with GDM? There were few studies on this issue currently. This study aimed to investigate the influence of age in adverse pregnancy outcomes in pregnant women with GDM.

Hebei Province is a populous province in northern China. The prevalence of GDM in Hebei Province was comparable to the national average, which ranged from 8.4 to 18.0%^3^. The database for our study was obtained from Hebei Province Maternal Near Miss Surveillance System (HBMNMSS) among 2015–2021, and the adverse pregnancy outcomes of pregnant women with GDM at different ages were analyzed.

## Material and methods

### Study population and data collection

**Figure 1:**
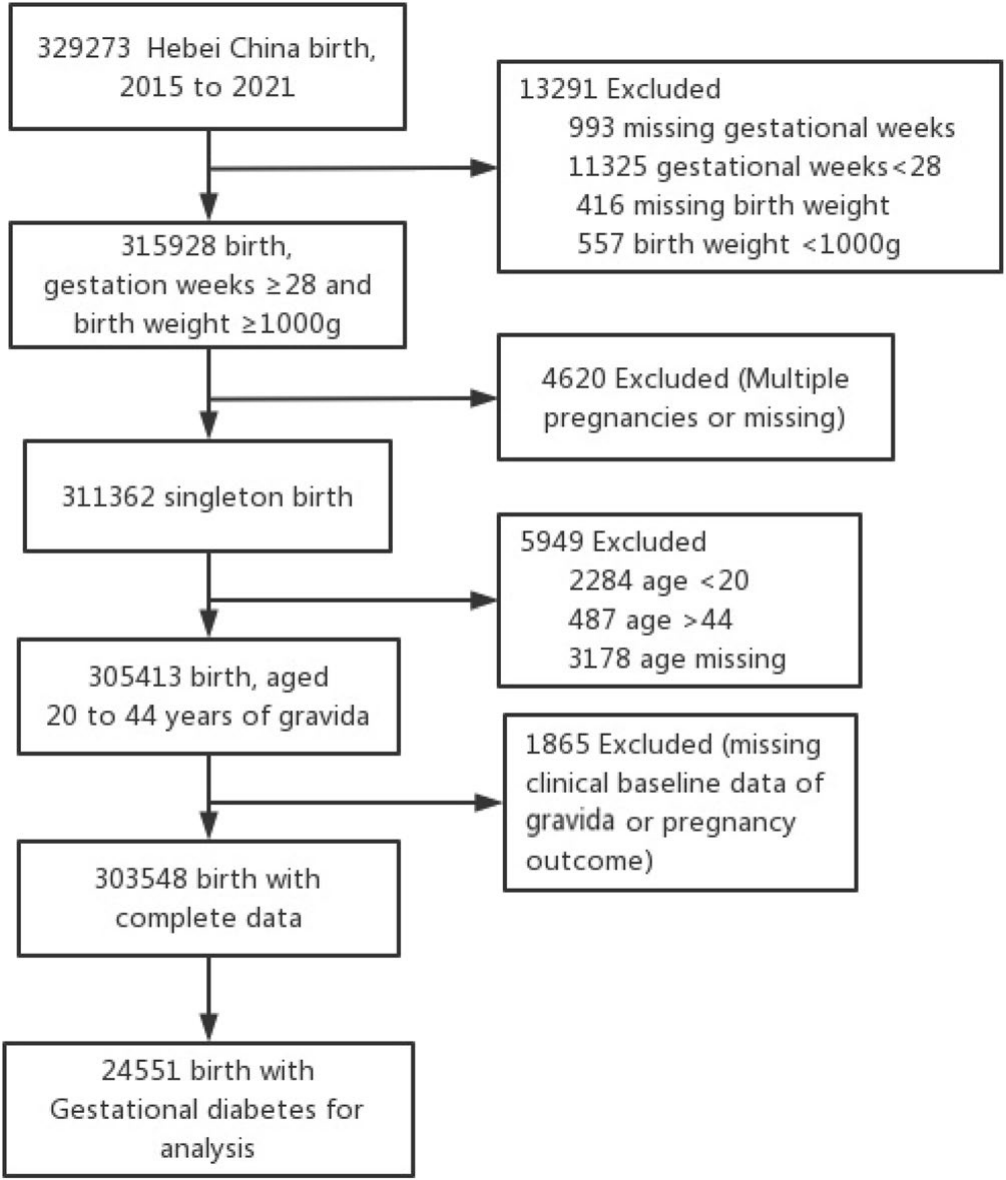
Selection of case flow diagram. The data was collected from Hebei Maternal Near Miss Surveillance System, which involved 22 hospitals in Hebei Province, China. There were 329,273 gravida from 2015 to 2021 and 24,551 pregnant women with GDM included in the research.

The data, which included 328,273 births, was collected in a random cluster sample from 22 hospitals (tertiary, secondary, and primary) in Hebei China from January 1, 2015 to December 31, 2021. Every year, more than 1000 infants are born at each of the hospitals. The data was collected by HBMNMSS, which was a sub-database of China's National Maternal Near Miss Surveillance System (NMNMSS). The database was entered and processed by special personnel, which was collected by trained doctors based on random stratified cluster sampling. We obtained authorization from Hebei Women and Children's Health Center to use the database. This study included singleton pregnant women with GDM aged 20–44 years, with a gestation week ≥ 28 weeks and a newborn weight ≥ 1000 g. Figure 1 depicts the flow chart of case enrollment. Finally, 24,551 pregnant women with GDM were enrolled. The adverse pregnancy outcomes of pregnant women with GDM at different age were analyzed. The 24,551 pregnant women with GDM were divided into five groups for analysis: 20–24 years old, 25–29 years old, 30–34 years old, 35–39 years old, and 40–44 years old. Between 24 and 28 weeks of pregnancy, oral glucose tolerance tests were performed on pregnant women. GDM was detected as fasting levels of 5.1 mmol/L, one-hour postprandial levels of 10.0 mmol/L, and two-hour postprandial levels of 8.5 mmol/L in oral glucose tolerance test^1^.

### Outcomes

The adverse pregnancy outcomes including maternal and infant outcomes. The adverse maternal outcomes were defined as: cesarean section delivery, abnormal fetal position, pre-eclampsia, anemia, placenta previa, placental abruption, uterine atony, and postpartum hemorrhage. The adverse infant outcomes were defined as: preterm birth, macrosomia, LGA, SGA, NICU admission, and neonatal death. Pregnant women aged ≥ 35 years was defined as AMA. The Kessner Index was used to determine that prenatal care was adequate, intermediate, and inadequate^13^. Placenta previa was described as placenta attached to the lower uterine segment lower than the fetal presentation, with the placental margin reaching or covering the cervical os after 28 weeks of gestation^14^. Pre-eclampsia was defined as hypertension (systolic blood pressure ≥ 140 mmHg or diastolic blood pressure ≥ 90 mmHg), proteinuria (≥ 300 mg/day), or liver, kidney functional failure, or nerve, blood system abnormalities after 20 weeks of gestation^15^. Anemia was defined as hemoglobin level ≤ 110 g/L. Placental abruption was defined as the separation of the placenta from the uterine wall while the fetus remained still in the uterine cavity after 20 weeks of gestation^16^. Postpartum hemorrhage was defined as blood loss ≥ 500 mL after vaginal birth or ≥ 1000 mL after caesarean delivery^17^. Preterm birth was defined as delivery from 28 to 36 + 6 weeks gestation^18^. Macrosomia was defined as birth weight ≥ 4000 g. Birth weight below the 10th percentile at each gestational week was classified as SGA, while birth weight over the 90th percentile at each gestational week was defined as LGA^19,20^.

### Statistical analysis

For qualitative data analysis, the Chi-square test was used. For normally distributed data, one-way analysis of variance (ANOVA) was used, and for non-normally distributed variables, the Kruskal–Wallis H test was used. The influence of age on the risk of adverse pregnancy outcomes in GDM pregnant women was analyzed using univariable and multivariable unconditional logistic regression analysis. Prenatal care, maternal education, gravidity, parity, delivery place, gestational week, and previous cesarean delivery were adjusted using multivariable logistic regression. The reference group was women aged 25–29 years. The result was presented as odds ratio (OR) or adjusted odds ratio (aOR) and 95% confidence interval (CI). *P* < 0.05 was considered as statistically significant. SPSS Version 17.0 and GraphPad Prism 8.0.1 statistical tools were used.

### Ethics approval

The data obtained from the NMNMSS were anonymised and did not infringe on patients’ privacy, therefore subjects could not be identified**.** The ethics committee of Hebei Reproductive Health Hospital and Hebei Center for Women and Children’s Health waived the need for informed consent from patients due to the retrospective nature of the study. All methods in our study were approved by Hebei Women and Children’s Health Center (Supplementary Material 2).

## Results

**Figure 2:**
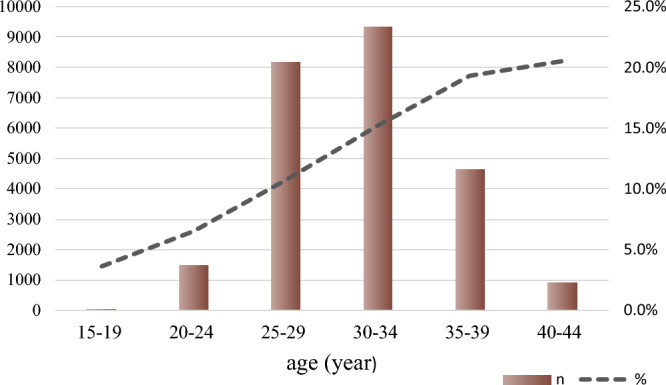
GDM number and prevalence in Hebei, China, from 2015 to 2021. The graph depicts that the prevalence of GDM rose with maternal age. The number is shown by a bar chart, while the rate is shown by a line chart.

Figure 1 shows the case screening process from HBMNMSS. From 2015 to 2021, data on 328,273 pregnant women were collected from 22 hospitals within 10 areas in Hebei, China. A total of 308,175 pregnant women were selected, and 24,551 pregnant women with GDM were analyzed. From 2015 to 2021, the prevalence of GDM in pregnant women was 8.1% in our study. Pregnant women's age was linked to an increased prevalence of GDM. GDM was found in around 20% of pregnant women aged over 35 years, and in only 3.6% of pregnant women aged 15–19 years, with a total of 52 cases (Fig. 2). In this study, 24,548 GDM pregnant women aged 20–44 years were analyzed.

The clinical baseline characteristics of 24,548 pregnant women with GDM are shown in Table 1. There were significant differences between primipara and multipara pregnant women with GDM at different ages (*p* < 0.001). The frequency of primipara was higher in pregnant women with GDM aged 20–24 years and 25–29 years, with 82.5% and 64.4%, respectively. The majority of AMA with GDM were multipara, and the rate of multipara in pregnant women with GDM aged 35–39 years and 40–44 years was 86.8% and 90.5%, respectively. Pregnant women with GDM aged 35–39 years and 40–44 years with less than 6 years of education had a higher ratio of 2.9% and 3.6%, respectively (*P* < 0.001). The frequency of pregnant women with GDM who were delivered in primary hospitals was associated with maternal age. In primary hospitals, the delivery rate of pregnant women with GDM of the five groups was 66.5%, 73.7%, 76.5%, 78.9%, and 82.1%, respectively (*P* < 0.001). The rate of pregnant women with GDM aged 40–44 years who had inadequate prenatal care was 7.0% (*P* < 0.001). There were no significant differences in the marital status and sex ratio of newborn infants in different age groups of pregnant women with GDM (*P* > 0.05).

**Table 1:** Clinical baseline data of 24,548 pregnant women with GDM in Hebei Province, China, from 2015 to 2021.

	20–24 years (*n* = 1489)	25–29 years (*n* = 8177)	30–34 years (*n* = 9323)	35–39 years (*n* = 4646)	40–44 years (*n* = 916)	*F/χ*^2^	*P*
Primipara	1229 (82.5)	5268 (64.4)	3017 (32.4)	611 (13.2)	87 (9.5)		
Multipara	260 (17.5)	2909 (35.6)	6306 (67.6)	4035 (86.8)	829 (90.5)	5044.73	< 0.001
History of cesarean delivery	118 (7.9)	1537 (18.8)	3536 (37.9)	2368 (51.0)	429 (46.8)	2042.03	< 0.001
Marital Status							
Married	1483 (99.6)	8144 (99.6)	9287 (99.6)	4627 (99.6)	912 (99.6)		
Single	6 (0.4)	33 (0.4)	36 (0.4)	19 (0.4)	4 (0.4)	0.09	0.999
Education (year)							
≤ 6	17 (2.1)	73 (1.4)	103 (1.6)	90 (2.9)	28 (3.6)		
7–9	307 (9.1)	1048 (31.1)	1216 (36.1)	665 (19.7)	134 (4.0)		
10–12	0 (0.0)	0 (0.0)	0 (0.0)	1 (0.0)	188 (24.2)		
> 12	489 (60.1)	4193 (78.9)	4925 (78.9)	2374 (75.8)	427 (55.0)	3976.78	< 0.001
Delivery place							
Tertiary Hospita	990 (66.5)	6030 (73.7)	7132 (76.5)	3708 (79.8)	752 (82.1)		
Secondary Hospital	499 (33.5)	2144 (26.2)	2190 (23.5)	938 (20.2)	164 (17.9)		
Primary Hospital	0 (0.0)	3 (0.0)	1 (0.0)	0 (0.0)	0 (0.0)	155.34	< 0.001
Prenatal care							
Adequate	580 (41.6)	3728 (48.3)	4442 (50.2)	2028 (46.4)	374 (43.6)		
Intermediate	742 (53.2)	3664 (47.5)	4035 (45.6)	2106 (48.2)	424 (49.4)		
Inadequate	72 (5.2)	320 (4.1)	378 (4.3)	233 (5.3)	60 (7.0)	66.29	< 0.001
Gestational Age (weeks)	38.7 (1.7)	38.7 (1.6)	38.4 (1.6)	38.2 (1.6)	37.8 (1.6)	112.74	< 0.001
Neonatal Sex							
Male	728 (48.9)	4174 (51.0)	4802 (51.5)	2411 (51.9)	489 (53.4)		
Female	761 (51.1)	4003 (49.0)	4520 (48.5)	2235 (48.1)	427 (46.6)	7.70	0.463

Continuous variables are presented as‾χ ± S, counting data variables are presented as n (%). *p* < 0.05 was considered statistically significant. GDM, gestational diabetes mellitus.

**Figure 3:**
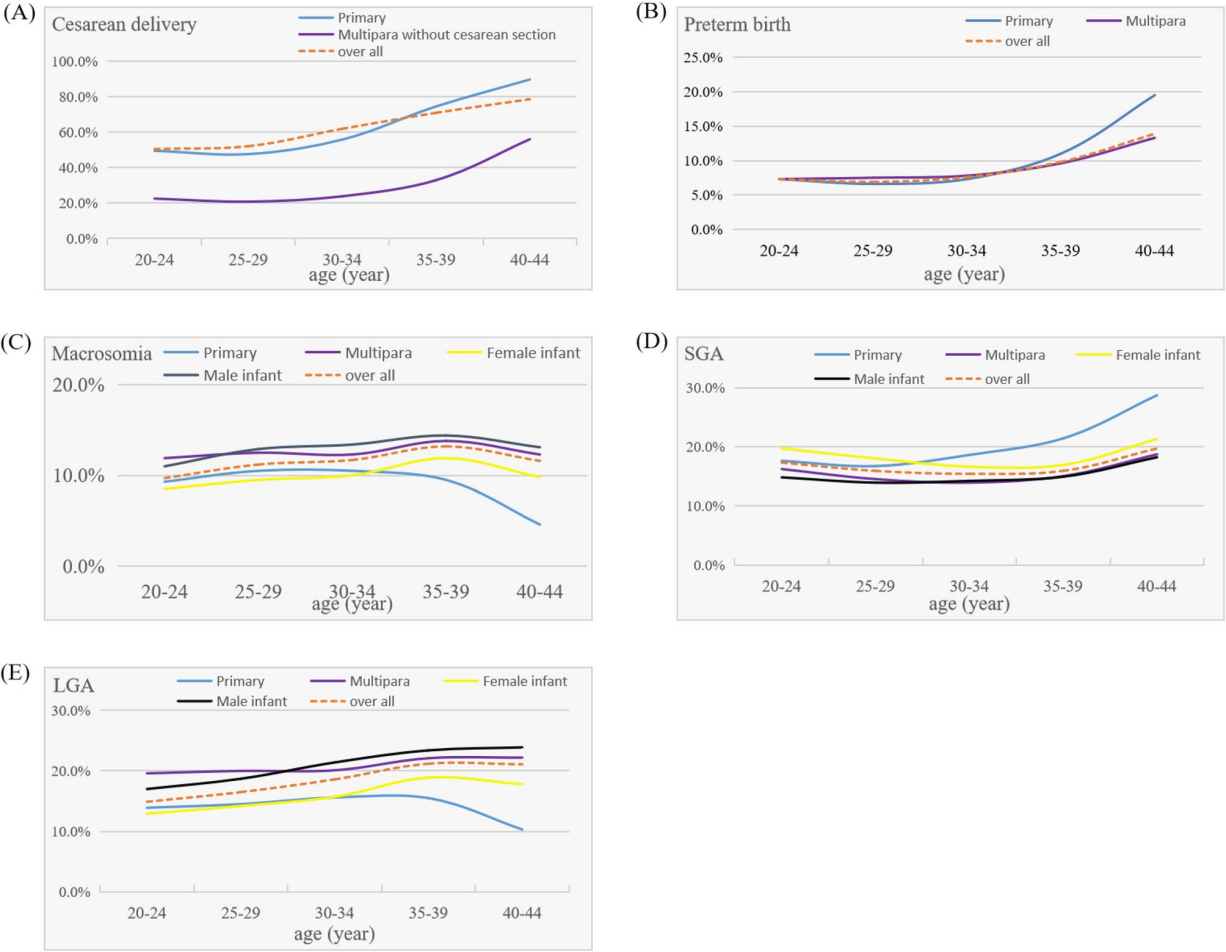
Adverse pregnancy outcomes of pregnant women with GDM aged 20–44 years. Figures (**A**)–(**E**) show the link between maternal age and the frequency of cesarean delivery, preterm birth, macrosomia, small for gestational age (SGA), and large for gestational age (LGA).

As shown in Table 2 and Fig. 3(A), the total cesarean delivery rate was strongly associated with maternal age in pregnant women with GDM aged 20–44 years. Furthermore, the cesarean delivery rate of primipara and multipara without previous cesarean section pregnant women with GDM aged 20–44 years was strongly related to maternal age (*P* < 0.001). The cesarean delivery rate among pregnant women with GDM in the five groups was 50.5%, 52.1%, 61.9%, 70.9%, and 78.6%, respectively. The cesarean delivery rate was lower in primipara (47.6%) and multipara without previous cesarean section (20.8%) women with GDM aged 25–29 years. Whereas primipara (89.7%) and multipara without previous cesarean section (56.0%) with GDM aged 40–44 years had the higher cesarean delivery rates. The prevalence of placenta previa, abnormal fetal position, and SGA was the highest in pregnant women with GDM aged 40–44 years, at 1.0%, 4.6%, and 19.7%, respectively. In primipara with GDM aged 40–44 years, the prevalence of SGA was 28.7% (*P* < 0.05). Pregnant women with GDM aged 25–29 years had the lowest rate of pre-eclampsia (4.5%) and preterm birth (6.9%), and who aged 40–44 years had the highest rate of pre-eclampsia (7.9%) and preterm birth (13.9%) (*P* < 0.001). Especially, the prevalence of preterm birth in primipara with GDM aged 40–44 years was 19.5% (*P* < 0.05) (Fig. 3B). Pregnant women with GDM aged 20–24 years had the lowest frequency of macrosomia (9.7%), and who aged 35–39 years had the highest rate (13.2%) (*P* < 0.01) (Fig. 3C). The primipara with GDM aged 40–44 years had the highest rate of SGA, which was 28.7% (*P* < 0.001) (Fig. 3D). The overall prevalence of LGA was associated with maternal age in pregnant women with GDM aged 20–44 years, however, the rate of LGA in primipara who aged 25–29 years was the lowest, at 10.3% (*P* < 0.001) (Fig. 3E). In pregnant women with GDM aged 40–44 years, the frequency of NICU admission and neonatal death increased, although the difference was not statistically significant (*P* > 0.05).

**Table 2:** Adverse pregnancy outcomes of pregnant women with GDM aged 20–44 years in Hebei Province, China, from 2015 to 2021.

Outcomes	20–24 years (*n* = 1489)	25–29 years (*n* = 8177)	30–34 years (*n* = 9323)	35–39 years (*n* = 4646)	40–44 years (*n* = 916)	*χ*^2^	*P*
Cesarean delivery	752 (50.5)	4261 (52.1)	5771 (61.9)	3292 (70.9)	720 (78.6)	647.32	< 0.001
Placenta previa	5 (0.3)	24 (0.3)	46 (0.5)	47 (1.0)	9 (1.0)	33.74	< 0.001
Pre-eclampsia	86 (5.8)	365 (4.5)	431 (4.6)	241 (5.2)	72 (7.9)	25.48	< 0.001
Anemia	684 (45.9)	3228 (39.5)	3642 (39.1)	1772 (38.1)	335 (36.6)	33.25	< 0.001
Placental abruption	3 (0.2)	23 (0.3)	27 (0.3)	21 (0.5)	5 (0.5)	5.28	0.26
Uterine atony	41 (2.8)	198 (2.4)	206 (2.2)	102 (2.2)	23 (2.5)	2.57	0.632
Postpartum hemorrhage	35 (2.4)	189 (2.3)	192 (2.1)	97 (2.1)	19 (2.1)	1.73	0.785
Retained placenta	2 (0.1)	13 (0.2)	15 (0.2)	12 (0.3)	3 (0.3)	3.19	0.527
Birth canal injury	1 (0.1)	17 (0.2)	12 (0.1)	7 (0.2)	1 (0.1)	2.77	0.597
Abnormal fetal position	44 (3.0)	221 (2.7)	253 (2.7)	161 (3.5)	42 (4.6)	16.49	0.002
Preterm birth	109 (7.3)	566 (6.9)	713 (7.6)	454 (9.8)	127 (13.9)	77.85	< 0.001
Macrosomia	145 (9.7)	918 (11.2)	1094 (11.7)	613 (13.2)	106 (11.6)	17.36	0.002
LGA	222 (14.9)	1349 (16.5)	1738 (18.6)	986 (21.2)	193 (21.1)	61.24	< 0.001
SGA	258 (17.3)	1303 (15.9)	1437 (15.4)	737 (15.9)	180 (19.7)	13.50	0.009
NICU admission	17 (1.1)	53 (0.6)	88 (0.9)	46 (1.0)	11 (1.2)	8.41	0.078
Neonatal death	0 (0.0)	7 (0.1)	9 (0.1)	2 (0.0)	2 (0.2)	4.44	0.349

Counting data variables are presented as n (%). *P* < 0.05 was considered statistically significant. *GDM* gestational diabetes mellitus, *LGA* large for gestational age, *SGA* small for gestational age, *NICU* neonatal intensive care unit.

**Figure 4:**
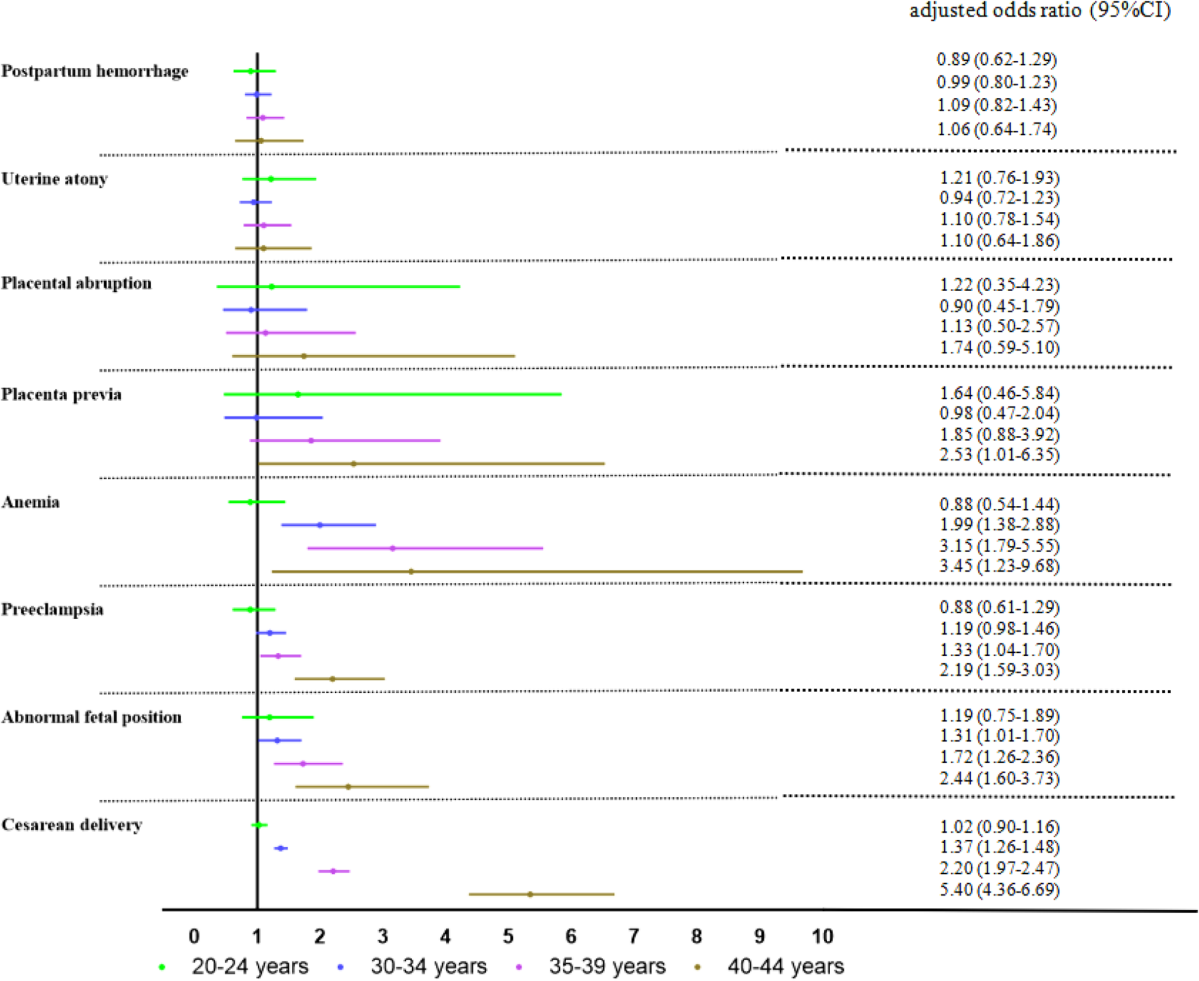
Forest plots of adjusted odds ratio for adverse maternal outcomes with GDM at different ages. Maternal aged 25–29 years as the reference group. The adjusted odds ratio and 95%CI by multiple logistic regression model after adjusted by prenatal care, maternal education, gravidity, parity, delivery place, gestational week, and previous cesarean delivery were adjusted using multivariable logistic regression.

Compared with GDM women aged 25–29 years, GDM women aged 35–44 years had a significantly higher risk of cesarean delivery, abnormal fetal position, pre-eclampsia, anemia, macrosomia, and LGA (*P* < 0.05). There had the highest risk of cesarean delivery (aOR: 5.40, 95% CI 4.36–6.69), abnormal fetal position (aOR: 2.44, 95% CI 1.60–3.73), pre-eclampsia (aOR: 2.19, 95% CI 1.59–3.03), anemia (aOR: 3.45, 95% CI 1.23–9.68), placenta previa (aOR: 2.53, 95% CI 1.01–6.35) in GDM women aged 40–44 years. Compared with GDM women aged 25–29 years, GDM women aged 20–24 years had a significantly higher risk of pre-eclampsia (aOR: 1.31, 95% CI 1.03–1.17) and anemia (aOR: 1.30, 95% CI 1.17–1.46), but there were no statistically significant difference in the effects of re-eclampsia (aOR: 0.88, 95% CI 0.61–1.29) and anemia (aOR: 0.88, 95% CI 0.54–1.44) after adjusting the confounding factors. There was no difference in the risk of placental abruption, uterine atony, and postpartum hemorrhage after adjusted for confounding factors (Fig. 4). For more information, see supplementary Table 1.

**Figure 5:**
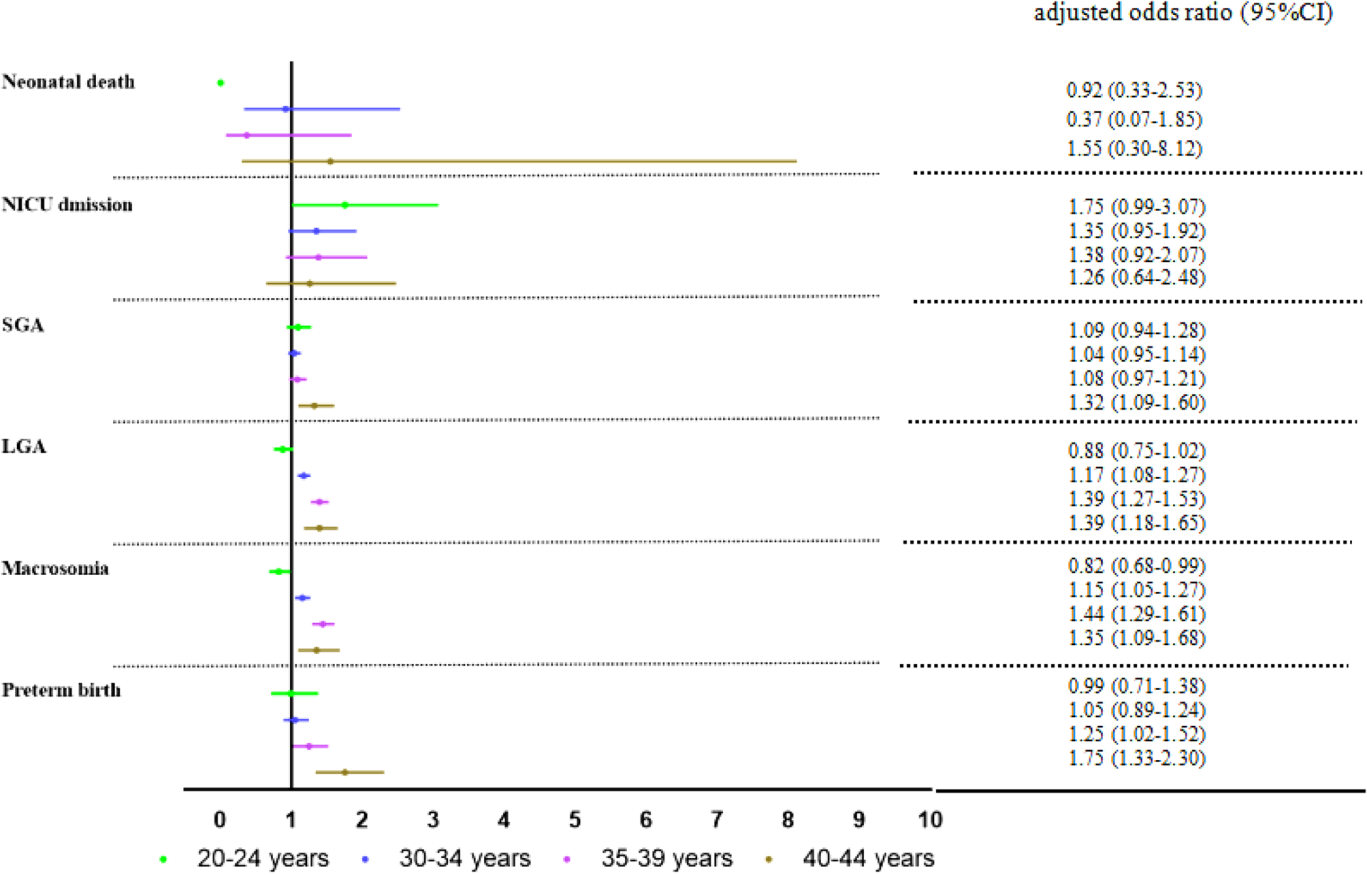
Forest plots of adjusted odds ratio for adverse infant outcomes with GDM at different ages. Maternal aged 25–29 years as the reference group. The adjusted odds ratio and 95%CI by multiple logistic regression model after adjusted by prenatal care, maternal education, gravidity, parity, delivery place, gestational week, and previous cesarean delivery were adjusted using multivariable logistic regression.

Compared with GDM women aged 25–29 years, GDM women aged 30–44 years had a significant higher risk of macrosomia and LGA, GDM women aged 35–44 years had a significant higher risk of preterm birth, and who aged 40–44 years had a significant higher risk of SGA (*P* < 0.05). Compared with GDM women aged 25–29 years, GDM women aged 20–24 years had a significant lower risk of macrosomia (aOR: 0.82, 95% CI 0.68–0.99). There was no difference in the risk of NICU admission and neonatal death after adjusted for confounding factors (Fig. 5). For more information, see supplementary Table 2.

## Discussion

This study showed a significantly higher prevalence of GDM in AMA, and it is critical to focus on the negative pregnancy outcomes of AMA with GDM.

This study found that the prevalence of GDM in AMA was significantly higher, in which was around 20%, that was similar to the result in mainland China^3^. The metabolic dysfunctions were caused by abnormal pancreas islet function of pregnant women with AMA which could induce chronic diseases during pregnancy^21^. In this study, we noticed that the incidence of adverse pregnancy outcomes such as pre-eclampsia, uterine atony, abnormal fetal position, preterm birth, SGA, and NICU admission was lower in pregnant women with GDM aged 25–29 years, so that this group was selected as the reference group.

The World Health Organization (WHO) advises limiting the cesarean delivery rate between 10 and 15%^22^. From 2008 to 2014, the prevalence of cesarean delivery ranged from 28.8 to 34.9% in China, which was greater than the WHO recommendation, and it was continuously growing^23^. The prevalence of cesarean delivery has been shown to be strongly connected with maternal age, and pregnant women with AMA and GDM were risk factors for cesarean delivery^12,21^. The cesarean delivery rate in pregnant women with AMA ranged from 24.4 to 74.1%, and from 50.8 to 59.9% in pregnant women with GDM^12,24^. Our study had the similar results of the cesarean delivery rate, AMA with GDM was more than 70%, in which primipara had the highest cesarean delivery rate (> 90%), multipara without previous caesarean section who aged over 40 years had a rate of more than 50%. The risk of cesarean delivery in pregnant women with GDM was found to be positively correlated with AMA in this study. Pregnant women with GDM aged over 35 years had twofold increased risk of cesarean delivery compared to who aged 25–29 years. Furthermore, the risk of cesarean delivery was fivefold higher in pregnant women with GDM aged over 40 years than in who aged 25–29 years. Insufficient perfusion of the uterus and placenta caused by vascular dysfunction of pregnant women with AMA would increase the risks of pregnancy complications, which increased the risk of cesarean delivery^25^.

This study found that AMA was an independent risk factor for anemia and pre-eclampsia in pregnant women with GDM. This was similar to the previous study, that inadequate prenatal care and the lack of professional nutrition advice in AMA during pregnancy were linked to a rise in the prevalence of anemia^26^. For the reasons mentioned above, AMA with GDM had an elevated risk of anemia. The endothelial dysfunction, dyslipidemia, and inflammation of pregnant women with AMA increased the risk of pre-eclampsia^27^.

This study found that AMA was an independent risk factor for anemia and pre-eclampsia in pregnant women with GDM. This was similar to the previous study, that AMA was a risk factor for anemia and pre-eclampsia^25^. Several studies have shown that inadequate prenatal care and the lack of professional nutrition advice during pregnancy were linked to a rise in the prevalence of anemia^26^. For the reasons mentioned above, AMA with GDM had an elevated risk of anemia. And it was considered that inadequate prenatal care and the endothelial dysfunction, dyslipidemia, and inflammation of pregnant women with AMA increased the risk of pre-eclampsia^27^. This may explain this study’s result, that AMA with GDM was an independent risk factor for pre-eclampsia.

The prevalence of preterm birth in pregnant women with GDM was found to be positively associated with AMA in this study, and who aged 40–44 years was found to be an independent risk factor for preterm birth. The previous findings suggested that pregnant women with AMA and GDM were at a higher risk of preterm birth^25^, which were consistent with the findings of this study. This was due to the high incidence of pregnancy complications in GDM and AMA, which was related the risk increased of preterm birth^28^. Pregnant women with GDM had a higher risk of spontaneous preterm birth^28^, and pre-eclampsia had a higher risk of medically required preterm birth^29^.

This study found that pregnant women with GDM aged 20–24 years was a protective factor for macrosomia, and AMA with GDM was a risk factor for macrosomia and LGA. This was consistent with the previous research findings, which found that macrosomia and LGA were always more common in pregnant women with GDM^5,30^, and AMA was also a risk factor for macrosomia and LGA^12,28^. Pregnant women with AMA and GDM was lower for metabolize glucose, and excessive placental transport of glucose and other nutrients from pregnant woman to fetus caused abnormal fetal growth, including macrosomia and LGA^30^. The length of the biparietal diameter of macrosomia increased, and the fetal position appeared abnormally^31^, and pregnant women with AMA were at increased risk of breech presentation^32^. These findings were consistent to the result of this study, that was, AMA with GDM had the higher risk of abnormal fetal position.

This study was a multi-center cohort study which used data from 22 hospitals in Hebei, China, and covered ten cities. The number of pregnant women with AMA and GDM had significantly rise as China’s twice-birth in 2016 and will even increasing after third-birth policies in 2021. Our study investigated the impact of age on adverse pregnancy outcomes in GDM pregnant women, which was beneficial in managing pregnant women with GDM and AMA. However, because this was a retrospective study, it had certain limitations. The data did not include information on body mass index, pregnancy weight increase, blood glucose levels during pregnancy, and blood glucose management (diet, exercise or insulin treatment), which might be attributed to the results’ bias.

## Conclusion

In pregnant women with GDM, AMA was an independent risk factor for caesarean section, anemia, abnormal fetal position, pre-eclampsia, macrosomia, and LGA. Pregnant women with GDM aged over 40 years was an independent risk factor for SGA. This shows the necessary of paying greater attention to GDM with AMA and improving prenatal care to enhance pregnancy outcomes of them.

### Supplementary Information

Supplementary Table 1.

Supplementary Information 2.

## Data Availability

Data is available from the corresponding author on a reasonable request.
