# Supplementary material for: Risk of adverse pregnancy outcomes in pregnant women with gestational diabetes mellitus by age: a multicentric cohort study in Hebei, China
Source: Sci Rep. 2024 Jan 8;14:807. doi: 10.1038/s41598-023-49916-2 (PMC10774329; doi:10.1038/s41598-023-49916-2)
Supplement: Supplementary file 1 — Supplementary Table 1. [file 41598_2023_49916_MOESM1_ESM.docx]

**Supplementary Table 1** Subgroup analysis of adverse pregnancy outcomes in GDM aged 20-44 in Hebei Province, China, from 2015 to 2021

| Outcomes | 20-24 years | 25-29 years | 30-34 years | 35-39 years | 40-44 years | Total | *χ*2 | *P* |
| --- | --- | --- | --- | --- | --- | --- | --- | --- |
|  | (*n*=1489) | (*n*=8177) | (*n*=9323) | (*n*=4646) | (*n*=916) | (n=24551) |  |  |
| Cesarean delivery |  |  |  |  |  |  |  |  |
| Over all | 752 (50.5) | 4261 (52.1) | 5771 (61.9) | 3292 (70.9) | 720 (78.6) | 14796 (60.3) | 647.32 | <0.001 |
| Primary | 608 (49.5) | 2508 (47.6) | 1683 (55.8) | 455 (74.5) | 78 (89.7) | 5332 (52.2) | 235.50 | <0.001 |
| Multipara without cesarean section | 32 (22.5) | 286 (20.8) | 659 (23.8) | 549 (32.9) | 224 (56.0) | 1750 (27.6) | 259.99 | <0.001 |
| Preterm birth |  |  |  |  |  |  |  |  |
| Over all | 109 (7.3) | 566 (6.9) | 713 (7.6) | 454 (9.8) | 127 (13.9) | 1969 (8.0) | 77.85 | <0.001 |
| Primary | 90 (7.3) | 348 (6.6) | 221 (7.3) | 67 (11) | 17 (19.5) | 743 (7.3) | 35.25 | <0.001 |
| Multipara | 19 (7.3) | 218 (7.5) | 492 (7.8) | 387 (9.6) | 110 (13.3) | 1226 (8.6) | 38.38 | <0.001 |
| Macrosomia |  |  |  |  |  |  |  |  |
| Over all | 145 (9.7) | 918 (11.2) | 1094 (11.7) | 613 (13.2) | 106 (11.6) | 2876 (11.7) | 17.36 | <0.01 |
| Primary | 114 (9.3) | 554 (10.5) | 317 (10.5) | 58 (9.5) | 4 (4.6) | 1047 (10.3) | 5.29 | 0.259 |
| Multipara | 31 (11.9) | 364 (12.5) | 777 (12.3) | 555 (13.8) | 102 (12.3) | 1829 (12.8) | 5.15 | 0.272 |
| Female infant | 65 (8.5) | 380 (9.5) | 451 (10.0) | 266 (11.9) | 42 (9.8) | 1204 (10.1) | 11.77 | <0.05 |
| Male infant | 80 (11.0) | 538 (12.9) | 634 (13.4) | 347 (14.4) | 64 (13.1) | 1672 (13.3) | 6.53 | 0.163 |
| SGA |  |  |  |  |  |  |  |  |
| Over all | 258 (17.3) | 1303 (15.9) | 1437 (15.4) | 737 (15.9) | 180 (19.7) | 3915 (15.9) | 13.50 | <0.01 |
| Primary | 216 (17.6) | 880 (16.7) | 560 (18.6) | 131 (21.4) | 25 (28.7) | 1812 (17.7) | 18.23 | <0.01 |
| Multipara | 42 (16.2) | 423 (14.5) | 877 (13.9) | 606 (15.0) | 155 (18.7) | 2103 (14.7) | 14.56 | <0.01 |
| Female infant | 150 (19.7) | 722 (18.0) | 752 (16.6) | 378 (16.9) | 91 (21.3) | 2093 (17.5) | 10.52 | 0.032 |
| Male infant | 108 (14.8) | 581 (13.9) | 684 (14.2) | 359 (14.9) | 89 (18.2) | 1821 (14.4) | 7.15 | 0.128 |
| LGA |  |  |  |  |  |  |  |  |
| Over all | 222 (14.9) | 1349 (16.5) | 1738 (18.6) | 986 (21.2) | 193 (21.1) | 4488 (18.3) | 61.24 | <0.001 |
| Primary | 171 (13.9) | 766 (14.5) | 471 (15.6) | 95 (15.5) | 9 (10.3) | 1512 (14.8) | 4.26 | 0.372 |
| Multipara | 51 (19.6) | 583 (20.0) | 1267 (20.1) | 891 (22.1) | 184 (22.2) | 2976 (20.8) | 8.16 | 0.086 |
| Female infant | 98 (12.9) | 569 (14.2) | 708 (15.7) | 423 (18.9) | 76 (17.8) | 1874 (15.7) | 30.28 | <0.001 |
| Male infant | 124 (17.0) | 780 (18.7) | 1030 (21.4) | 563 (23.4) | 117 (23.9) | 2614 (20.7) | 31.28 | <0.001 |

Counting data variables are presented as n (%). P < 0.05 was considered statistically significant. GDM, gestational diabetes mellitus; LGA, large for gestational age; SGA, small for gestational age; NICU, neonatal intensive care unit.

**Supplementary Table 2** Odds ratio and 95%CI of adverse maternal outcomes in pregnant women with GDM at different ages

|  | Maternal age  (years) | *OR* (95%*CI*) | *P* | a*OR* (95%*CI*) | *P* |
| --- | --- | --- | --- | --- | --- |
| Cesarean delivery | 20-24 | 0.94 (0.84-1.05) | 0.254 | 1.02 (0.90-1.16) | 0.743 |
|  | 30-34 | 1.49 (1.41-1.59) | <0.001 | 1.37 (1.26-1.48) | <0.001 |
|  | 35-39 | 2.23 (2.07-2.41) | <0.001 | 2.20 (1.97-2.47) | <0.001 |
|  | 40-44 | 3.38 (2.89-3.98) | <0.001 | 5.40 (4.36-6.69) | <0.001 |
| Abnormal fetal position | 20-24 | 1.10 (0.79-1.52) | 0.584 | 1.19 (0.75-1.89) | 0.455 |
|  | 30-34 | 1.00 (0.84-1.21) | 0.964 | 1.31 (1.01-1.70) | 0.039 |
|  | 35-39 | 1.29 (1.05-1.59) | 0.015 | 1.72 (1.26-2.36) | 0.001 |
|  | 40-44 | 1.73 (1.24-2.42) | 0.001 | 2.44 (1.60-3.73) | <0.001 |
| Pre-eclampsia | 20-24 | 1.31 (1.03-1.67) | 0.028 | 0.88 (0.61-1.29) | 0.514 |
|  | 30-34 | 1.04 (0.90-1.20) | 0.614 | 1.19 (0.98-1.46) | 0.081 |
|  | 35-39 | 1.17 (0.99-1.38) | 0.064 | 1.33 (1.04-1.70) | 0.023 |
|  | 40-44 | 1.83 (1.40-2.37) | <0.001 | 2.19 (1.59-3.03) | <0.001 |
| Anemia | 20-24 | 1.30 (1.17-1.46) | <0.001 | 0.88 (0.54-1.44) | 0.617 |
|  | 30-34 | 0.98 (0.93-1.04) | 0.578 | 1.99 (1.38-2.88) | <0.001 |
|  | 35-39 | 0.95 (0.88-1.02) | 0.136 | 3.15 (1.79-5.55) | <0.001 |
|  | 40-44 | 0.88 (0.77-1.11) | 0.088 | 3.45 (1.23-9.68) | 0.019 |
| Placenta previa | 20-24 | 1.15 (0.44-3.01) | 0.784 | 1.64 (0.46-5.84) | 0.442 |
|  | 30-34 | 1.68 (1.03-2.76) | 0.039 | 0.98 (0.47-2.04) | 0.956 |
|  | 35-39 | 3.47 (2.12-5.68) | <0.001 | 1.85 (0.88-3.92) | 0.107 |
|  | 40-44 | 3.37 (1.56-7.27) | 0.002 | 2.53 (1.01-6.35) | 0.048 |
| Placental abruption | 20-24 | 0.72 (0.22-2.39) | 0.586 | 1.22 (0.35-4.23) | 0.750 |
|  | 30-34 | 1.03 (0.59-1.80) | 0.918 | 0.90 (0.45-1.79) | 0.760 |
|  | 35-39 | 1.61 (0.89-2.91) | 0.115 | 1.13 (0.50-2.57) | 0.771 |
|  | 40-44 | 1.95 (0.74-5.13) | 0.178 | 1.74 (0.59-5.10) | 0.315 |
| Uterine atony | 20-24 | 1.14 (0.81-1.61) | 0.448 | 1.21 (0.76-1.93) | 0.421 |
|  | 30-34 | 0.91 (0.75-1.11) | 0.352 | 0.94 (0.72-1.23) | 0.640 |
|  | 35-39 | 0.91 (0.71-1.15) | 0.416 | 1.10 (0.78-1.54) | 0.594 |
|  | 40-44 | 1.04 (0.67-1.61) | 0.868 | 1.10 (0.64-1.86) | 0.736 |
| Postpartum hemorrhage | 20-24 | 1.02 (0.71-1.47) | 0.924 | 0.89 (0.62-1.29) | 0.543 |
|  | 30-34 | 0.89 (0.73-1.09) | 0.255 | 0.99 (0.80-1.23) | 0.921 |
|  | 35-39 | 0.90 (0.70-1.15) | 0.409 | 1.09 (0.82-1.43) | 0.563 |
|  | 40-44 | 0.90 (0.56-1.44) | 0.648 | 1.06 (0.64-1.74) | 0.835 |

The adjusted odds ratio and 95%CI by multiple logistic regression model after adjusted by prenatal care, maternal education, gravidity, parity, delivery place, gestational week, and previous cesarean delivery were adjusted using multivariate logistic regression. Maternal aged 25-29 years as the reference group. GDM, gestational diabetes mellitus.

**Supplementary Table 3** Odds ratio and 95%CI of adverse infant outcomes in pregnant women with GDM at different ages

|  | Maternal age  (years) | *OR* (95%CI) | *P* | a*OR* (95%*CI*) | *P* |
| --- | --- | --- | --- | --- | --- |
| Preterm birth | 20-24 | 1.06 (0.86-1.31) | 0.579 | 0.99 (0.71-1.38) | 0.954 |
|  | 30-34 | 1.11 (0.99-1.25) | 0.066 | 1.05 (0.89-1.24) | 0.580 |
|  | 35-39 | 1.46 (1.28-1.66) | <0.001 | 1.25 (1.02-1.52) | 0.031 |
|  | 40-44 | 2.16 (1.762-2.66) | <0.001 | 1.75 (1.33-2.30) | <0.001 |
| Macrosomia | 20-24 | 0.85 (0.71-1.03) | 0.092 | 0.82 (0.68-0.99) | 0.038 |
|  | 30-34 | 1.05 (0.96-1.15) | 0.293 | 1.15 (1.05-1.27) | 0.003 |
|  | 35-39 | 1.20 (1.08-1.34) | 0.001 | 1.44 (1.29-1.61) | <0.001 |
|  | 40-44 | 1.04 (0.84-1.28) | 0.754 | 1.35 (1.09-1.68) | 0.007 |
| LGA | 20-24 | 0.89 (0.76-1.04) | 0.127 | 0.88 (0.75-1.02) | 0.097 |
|  | 30-34 | 1.16 (1.07-1.25) | <0.001 | 1.17 (1.08-1.27) | <0.001 |
|  | 35-39 | 1.36 (1.24-1.49) | <0.001 | 1.39 (1.27-1.53) | <0.001 |
|  | 40-44 | 1.35 (1.14-1.60) | <0.001 | 1.39 (1.18-1.65) | <0.001 |
| SGA | 20-24 | 1.11 (0.96-1.28) | 0.180 | 1.09 (0.94-1.28) | 0.265 |
|  | 30-34 | 0.96 (0.89-1.04) | 0.344 | 1.04 (0.95-1.14) | 0.428 |
|  | 35-39 | 1.00 (0.90-1.10) | 0.915 | 1.08 (0.97-1.21) | 0.173 |
|  | 40-44 | 1.29 (1.09-1.53) | 0.004 | 1.32 (1.09-1.60) | 0.004 |
| NICU admission | 20-24 | 1.77 (1.02-3.07) | 0.041 | 1.75 (0.99-3.07) | 0.051 |
|  | 30-34 | 1.46 (1.04-2.06) | 0.030 | 1.35 (0.95-1.92) | 0.092 |
|  | 35-39 | 1.53 (1.03-2.28) | 0.035 | 1.38 (0.92-2.07) | 0.124 |
|  | 40-44 | 1.86 (0.97-3.58) | 0.062 | 1.26 (0.64-2.48) | 0.509 |
| Neonatal death | 20-24 |  | 0.989 |  | 0.988 |
|  | 30-34 | 1.13 (0.42-3.03) | 0.811 | 0.92 (0.33-2.53) | 0.865 |
|  | 35-39 | 0.50 (0.10-2.42) | 0.391 | 0.37 (0.07-1.85) | 0.225 |
|  | 40-44 | 2.55 (0.53-12.31) | 0.243 | 1.55 (0.30-8.12) | 0.606 |

The adjusted odds ratio and 95%CI by multiple logistic regression model after adjusted by prenatal care, maternal education, gravidity, parity, delivery place, gestational week, and previous cesarean delivery were adjusted using multivariate logistic regression. Maternal aged 25-29 years as the reference group. GDM, gestational diabetes mellitus; LGA, large for gestational age; SGA, small for gestational age; NICU, neonatal intensive care unit.
