# Supplementary material for: Risk of adverse pregnancy outcomes in pregnant women with gestational diabetes mellitus by age: a multicentric cohort study in Hebei, China
Source: Sci Rep. 2024 Jan 8;14:807. doi: 10.1038/s41598-023-49916-2 (PMC10774329; doi:10.1038/s41598-023-49916-2)
Supplement: Supplementary file 2 — Supplementary Information 2. [file 41598_2023_49916_MOESM2_ESM.pdf]

Title: Study of perinatal medicine in Hebei province
PI Name: T I N G Z H A N G
Date : October 29, 2017
Protocol Number: 20171029-1

The data came from Hebei province Maternal Near Miss Surveillance System (HBMNMSS), which collected and managed by Hebei Women and Children's Health Center, and supported by the National Health Commission of the People's Republic of China.

Perinatal medicine is a new science that has been developed over the past 30 years. It is the study of fetal physiology and pathology, and the diagnosis and prevention of neonatal, pregnancy and maternal diseases. We will conduct a review of the data of all the pregnant women and their fetus, neonatus, babies,to find the risk factors, prediction, prognosis of adverse outcomes of maternal and infant, to improve outcomes of maternal and infant. The purpose of the study is to summarize the presentation, characteristics, management, prenatal diagnosis and outcome of perinatal diseases, before, during and after pregnancy.

The research dose not involve an intervention and/or interaction with subjects for the collection of specimens or biological material, the data including health or clinical data, surveys, limited to the use of health / medical information.This study will provide important implication(s) for future service and researches. Information including but not limited to presenting signs, surgical decision making, care in the intensive care unit, well-being of the babies, outcome of the parturient, and demographic and obstetric information will be analyzed. For Data resulting from this research will be used for Publication or Fund application.

Data to be requested from the following time period (01/01/2013 - 01/01/2022). Direct identifiers, such as name and medical record number will not be recorded with the data. Patient related files will be either locked in cabinets or stored on password protected computer with access limited to study staff.

The study was agreed to implement by Hebei Women and Children's Health Center.
